# Voltage-Controlled Enzymes: The New *Janus* *Bifrons*

**DOI:** 10.3389/fphar.2012.00161

**Published:** 2012-09-13

**Authors:** Carlos A. Villalba-Galea

**Affiliations:** ^1^Department of Physiology and Biophysics, Virginia Commonwealth University School of MedicineRichmond, VA, USA

**Keywords:** voltage-sensitive phosphatases, Ci-VSP, sensing current, 3_10_ helix, VSD relaxation

## Abstract

The *Ciona intestinalis* voltage-sensitive phosphatase, Ci-VSP, was the first Voltage-controlled Enzyme (VEnz) proven to be under direct command of the membrane potential. The discovery of Ci-VSP conjugated voltage sensitivity and enzymatic activity in a single protein. These two facets of Ci-VSP activity have provided a unique model for studying how membrane potential is sensed by proteins and a novel mechanism for control of enzymatic activity. These facets make Ci-VSP a fascinating and versatile enzyme. Ci-VSP has a voltage sensing domain (VSD) that resembles those found in voltage-gated channels (VGC). The VSD resides in the N-terminus and is formed by four putative transmembrane segments. The fourth segment contains charged residues which are likely involved in voltage sensing. Ci-VSP produces sensing currents in response to changes in potential, within a defined range of voltages. Sensing currents are analogous to “gating” currents in VGC. As known, these latter proteins contain four VSDs which are entangled in a complex interaction with the pore domain – the effector domain in VGC. This complexity makes studying the basis of voltage sensing in VGC a difficult enterprise. In contrast, Ci-VSP is thought to be monomeric and its catalytic domain – the VSP’s effector domain – can be cleaved off without disrupting the basic electrical functioning of the VSD. For these reasons, VSPs are considered a great model for studying the activity of a VSD in isolation. Finally, VSPs are also phosphoinositide phosphatases. Phosphoinositides are signaling lipids found in eukaryotes and are involved in many processes, including modulation of VGC activity and regulation of cell proliferation. Understanding VSPs as enzymes has been the center of attention in recent years and several reviews has been dedicated to this area. Thus, this review will be focused instead on the other face of this true *Janus* *Bifrons* and recapitulate what is known about VSPs as electrically active proteins.

## Introduction

Voltage sensing phosphatases (VSP) are the first family of enzymes displaying a voltage sensing domain (VSD). The first member of the VSP family was described in 1999, when the human isoform TPTE (Transmembrane Phosphatase with Tensin homology) was reported as a testis-specific protein (Chen et al., 1999; Guipponi et al., 2001; Wu et al., 2001; Tapparel et al., 2003). In spite of the great similarities between the C-terminus of TPTE and members of the protein tyrosine phosphatases (PTP) family (Chen et al., 1999; Guipponi et al., 2000, 2001; Walker et al., 2001; Tapparel et al., 2003), no catalytic activity was – or has been – observed to be mediated by this protein.

Two years later, the findings of a second human VSP (Walker et al., 2001; Wu et al., 2001) and a murine VSP (Guipponi et al., 2001) were reported. In contrast to TPTE, the new human VSP (known as TPTE2 and originally named TPIP: TPTE and PTEN homologous Inositol lipid Phosphatase) displayed phosphoinositide phosphatase activity (Walker et al., 2001; Wu et al., 2001). Another difference between the human VSPs (Hs-VSP, where “Hs-” is for *Homo sapiens*) is that TPTE2 (hereafter Hs-VSP1) is also found expressed in stomach and brain, in addition to testis (Walker et al., 2001). To date, the physiological role of these proteins remains elusive. Likewise, whether or not Hs-VSPs are electrical active remains to be determined – so is the case for the murine VSP (Mm-VSP; known as mTpte). Nevertheless, it is arguably predicted that VSPs are involved in phosphoinositide signaling pathways, which are found in all eukaryotes (Di Paolo and De Camilli, 2006; Balla et al., 2009).

Since the discovery of Hs-VSPs, a number of VSPs have been found – or predicted to exist – in many species (Kumanovics et al., 2002; Sutton et al., 2012). The most conspicuous member of the family is Ci-VSP. This enzyme was isolated from the tunicate *Ciona intestinalis* – hence the acronym “Ci-.” In juvenile animals, Ci-VSP has a wide tissue distribution (Ogasawara et al., 2011); whereas it seems to be restricted to testis, neuronal tissues, and sperm in adults (Murata et al., 2005). In contrast to mammals VSPs, Ci-VSP displays robust electrical activity. Indeed, Ci-VSP was the first enzyme proven to be under direct control of the membrane potential (Murata et al., 2005; Murata and Okamura, 2007). Ci-VSP is one of the workhorses for research aimed at understanding the biophysical and biochemical features of the VSP family. In fact, our current understanding of the functioning of VSPs emerges from studies on this enzyme.

The physiological role of VSPs remains unclear. Ci-VSP and other catalytically active VSPs are phosphoinositides phosphatases (Walker et al., 2001; Murata et al., 2005; Murata and Okamura, 2007; Iwasaki et al., 2008; Halaszovich et al., 2009, 2012; Kohout et al., 2010; Ratzan et al., 2011; Kurokawa et al., 2012). As known, phosphoinositides are ubiquitous signaling lipids in eukaryotes (Di Paolo and De Camilli, 2006; Balla et al., 2009). Phosphoinositide signaling is central for a number of processes including development (Leslie and Downes, 2004; Di Paolo and De Camilli, 2006; Leslie et al., 2007, 2008; Balla et al., 2009), ion channels regulation (Suh and Hille, 2008; Logothetis et al., 2010), plasma membrane identity (Hammond et al., 2012), and others. Also, it has been shown that there is a correlation between changes in the membrane potential and regulation of cell proliferation and differentiation (Sundelacruz et al., 2009; Levin and Stevenson, 2012). Thus, VSPs constitute a potential direct link between electrical activity and development.

VSPs are homologs to PTEN, an enzyme critically involved in the control of cell growth and proliferation, as well as in cell differentiation (Leslie and Downes, 2002, 2004; Bai et al., 2004; Menager et al., 2004; Walker et al., 2004; Balla et al., 2005; Leslie et al., 2007, 2008; Endersby and Baker, 2008; Ooms et al., 2009; Arendt et al., 2010; Bunney and Katan, 2010; Davidson et al., 2010; Michailidis et al., 2011). PTEN is known as a *tumor suppressor* – disruption of its function is among the most common causes of cancer in humans (Li et al., 1997; Teng et al., 1997; Maehama and Dixon, 1998, 1999; Leslie and Downes, 2004; Bunney and Katan, 2010). PTEN and the catalytic domain of Ci-VSP display similar mechanisms for activation (Iwasaki et al., 2008; Villalba-Galea et al., 2009a; Kohout et al., 2010; Hobiger et al., 2012), share catalytic targets (Murata et al., 2005; Iwasaki et al., 2008; Halaszovich et al., 2009; Kohout et al., 2010; Lacroix et al., 2011), and have structures that resemble each other (Lee et al., 1999; Matsuda et al., 2011; Liu et al., 2012). Based on these similarities, a series of chimeras, made by attaching the VSD of Ci-VSP to PTEN, were proven to provide control by membrane potential on the activity of PTEN (Lacroix et al., 2011). This study demonstrated for the first time that a cytosolic enzyme can be engineered to become a Voltage-controlled Enzymes (VEnz) and, thus, be directly controlled by membrane potential. More recently, this approach has been used to study the activity of the catalytic domains of the chicken (*Gallus* *gallus*) VSP (Gg-VSP; Kurokawa et al., 2012) and the Hs-VSP1 (Halaszovich et al., 2012; Kurokawa et al., 2012).

Among enzymes, what is unique about VSPs is that the N-terminus forms a functional VSD controlling catalytic activity – at least in non-mammalian VSPs. In spite of this extraordinary characteristic, it is the C-terminus what has drawn the attention of many researchers in recent years. Presumably, a reason for this is that Ci-VSP displays high structural and functional homology with the tumor suppressor PTEN (Murata et al., 2005; Murata and Okamura, 2007; Iwasaki et al., 2008; Villalba-Galea et al., 2009a; Kohout et al., 2010; Lacroix et al., 2011; Hobiger et al., 2012; Liu et al., 2012). In fact, several review articles on this matter are available in the literature (Worby and Dixon, 2005; Okamura and Dixon, 2011; Villalba-Galea, 2012) and a number of crystal structures have been published recently (Matsuda et al., 2011; Liu et al., 2012). Arguably however, the most striking feature of VSPs is that the VSD controls catalytic activity. Thus, this review will be mainly focused on the electrical properties of VSPs.

## Sensing Currents

The VSD of Ci-VSP bears charged residues located within the membrane-embedded region of the protein. As for others VSD proteins, changes in magnitude and/or polarity of the electrical field across the plasma membrane can induce changes in the position of these charges, translating this displacement into conformational changes in the protein itself. This is the underlying process for voltage sensing (Bezanilla, 2005, 2008; Swartz, 2008).

**Figure 1:**
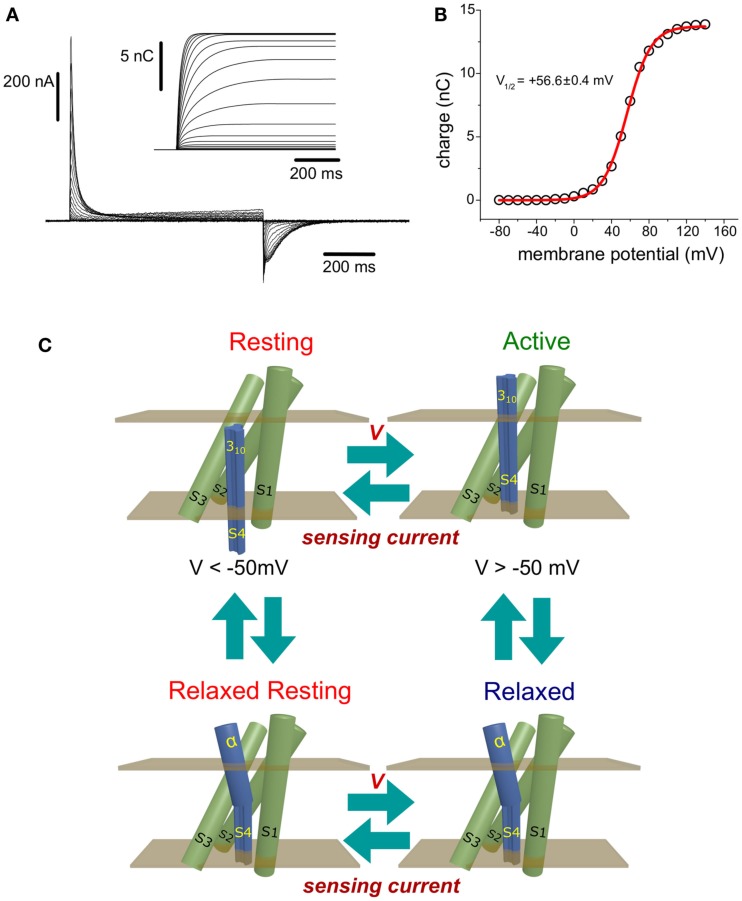
**(A)** Ci-VSP-C363S sensing currents recorded from *Xenopus* oocytes using the cut-open voltage clamp technique (Taglialatela et al., 1992). The holding potential (HP) was set to −60 mV, and ON-sensing currents were evoked by 800 ms-test pulses ranged −80 to +140 mV. OFF-sensing currents were recorded at −60 mV. Numerical integration of the ON-sensing currents (inset) was performed using a package developed by the author using the programming language Java. **(B)** Maximum (steady state) net charges are calculated by integration were plotted against the voltage applied during the corresponding test pulse. The charge (Q) vs. Potential (V) relationship was fitted to a Boltzmann distribution (see text). For this, particular example, the half-maximum potential fitted was +56.6 ± 0.4 mV. **(C)** Minimum scheme for description of the electrical behavior of the voltage sensing domain of Ci-VSP. At potentials below −50 mV, the VSD resides with high probability in the Resting state. Upon changes in the membrane potential to more positive voltages, sensing currents are observed as consequence of the movement of sensing charges leading the VSD into the active state. If the membrane potential is above +50 mV, a secondary, voltage-independent transition is observed following sensing currents. This process is called relaxation (see text) and promotes the population of the relaxed state. As described in the text, transitions between the resting and active state may occur while the S4 segment is in a 3_10_ helix conformation. However, transit into the relaxed states may be accompanied by a transformation of the upper part of the S4 segment into an α-helix. Finally, repolarization of the plasma membrane causes the return of the VSD to the resting state. This transition is achieved through a hypothetical relaxed resting state.

The movement of charged residues down the electrical gradient produces transient currents (Figure 1A). These currents are known as “sensing” currents. In voltage-gated channels (VGC), sensing currents are regarded as “gating” currents, since they are involved in the mechanism that opens and closes the “gate” for ion conduction (Bezanilla, 2005, 2008; Tombola et al., 2006). Thus, it is fair to say that “gating” currents were the first instance of sensing currents ever described.

Sensing currents are produced by the movement of VSD intrinsic charges across its membrane-embedded region. In the simplest case, VSD’s charges sojourn between two states, one called Resting state and another called Active state (Figure 1C, top). The Resting state corresponds to the most probable state found at resting membrane potentials – hence the name. In this condition, the plasma membrane is polarized at negative voltage. On the other hand, the VSD is more likely to be in the Active state as more positive the membrane potential is. The transition rates between Resting and Active states depend exponentially on the voltage across the membrane (Bezanilla, 2000). For a simple two-state model, the transition rate from the Resting to the Active state (α) is greater as the membrane potential is more positive, while the rate for the reverse transition (β) is lesser; for negative potentials, the opposite situation is observed. Thus, the probability of finding the VSD in the Active state (*P*_Active_) increases at more positive potentials and is given by the following equation: *P*_Active_ = α/(α +  β).

Usually, the action of changing the membrane potential to more positive values is referred to as “depolarization.” This term is inherited from classical electrophysiology in which conductances were evoked by driving the membrane potential toward 0 mV – not polarized membrane. However, in the case of VSPs, maximum activation is typically observed above +60 mV (Murata et al., 2005; Murata and Okamura, 2007; Hossain et al., 2008; Iwasaki et al., 2008; Villalba-Galea et al., 2009a; Ratzan et al., 2011). At these potentials, the membrane is positively polarized and the magnitude of the polarization is larger that the one observed at typical resting potentials. Thus, the term “depolarization” is unsuitable to describe the changes in potential that leads to activation of VSPs. Instead, the term antipolarization (anti-: from the greek αντí that means opposite) is a more accurate descriptor.

## The Nature of Sensing Currents

For the voltage-gated channel *Shaker*, it has been proposed that gating currents are composed by the sum of “shot”-like currents events (Sigg et al., 1994; Bezanilla, 2000). There is not reason to believe that VSPs behave differently. Thus, it can be assumed that, as in the case of *Shaker*, the transition of a single VSD from the Resting to the Active state produces an outward “shot”-like current as sensing charges move toward the extracellular space. In contrast, the transition from the Active to the Resting state produces an inward “shot”-like current as the sensing charges move in the opposite direction. For a large number of VSDs, the balance between these currents results in a net charge movement across the membrane, thus sensing currents.

When the membrane potential changes from negative to a more positive voltages, sensing currents are observed as outwardly rectifying currents. These currents are referred to as ON-sensing currents, since they are related to the activation of the VSD and phosphatase activity. Likewise, changes from positive to more negative potentials evoke inward sensing current, which are referred to as OFF-sensing currents, since they are related to the deactivation of the VSPs. The net charge movement (*Q*) at each potential can be determined by numerically integrating sensing currents (Figure 1A, inset). The relationship between *Q* and the membrane potential (*V*), known as *Q*−*V* relationship (Figure 1B, open circles), is typically described by one or the sum of two or more Boltzmann distributions $Q=Q_{\text{MAX}}/(1+{\text{e}}^{-zF(V-V_{1/2})/RT})$ (Figure 1B, red line). The parameters of these distributions are utilized to characterized voltage dependence of VSD proteins. One of the most commonly used parameters is the half-maximum potential (*V*_1/2_) that, in the case of a two-state model, defines at which potential the Resting and Active states are equally populated (Figure 1B). Other parameters for Boltzmann distributions are: *Q*_MAX_ which is the maximum charge that can be moved, *z* which is the apparent sensing charge, and *F*, *R*, and *T* which are the Faraday constant, the universal ideal gas constant, and *T* in temperature in Kelvin, respectively.

## Voltage Dependence of VSPs

For Ci-VSP, sensing currents typically become discernible at potentials above −50 mV, when holding the membrane at −60 mV. As describe above, *Q* increases as antipolarization increases and it reaches its maximum – it saturates – at potentials above +120 mV (Figure 1B). The typical *V*_1/2_ for the Ci-VSP *Q*−*V* relationship is around +55 mV (Hossain et al., 2008; Villalba-Galea et al., 2008; Figure 1B). Beside Ci-VSP, sensing currents have been only reported from the isoform isolated from *Danio* *rerio* (zebrafish). This VSP, known as Dr-VSP, shows a *V*_1/2_ around +96 mV (Hossain et al., 2008).

Three additional VSPs have been shown to be VEnz. These are two isoforms isolated from *Xenopus* *laevis* (Xl-VSP1 and Xl-VSP2) and one isoform isolated from *Xenopus* *tropicalis* (Xt-VSP; Ratzan et al., 2011). No sensing currents have been reported from these proteins. However, catalytic activity for Xl-VSP1 and Xl-VSP2 is observed at potential above −20 and 0 mV, respectively, reaching maximum around +60 mV (Ratzan et al., 2011). These observations suggest that Xl-VSPs have steeper voltage dependence than Ci-VSP.

An intriguing feature of VSP is that mutations in the catalytic domain, the effector domain of the VSD, have direct consequences on the electrical activity of the voltage sensor. Particularly, inactivation of Ci-VSP catalytic activity by mutating Cystein 363 to a serine (C363S) causes an apparent change in the dynamics of the VSD Ci-VSP. As reported from experiments using Two-Electrode Voltage Clamp Fluorometry (TE-VCF), the deactivation of the VSD is slower when the catalytic domains has been inactivated by introducing the mutation C363S (Kohout et al., 2010). Likewise, introduction of the equivalent mutation in Dr-VSP (C302S) slightly shifts the *V*_1/2_ from +97 to +107 mV (Hossain et al., 2008). The basis for these differences in the electrical properties is yet to be determined.

It has also been shown that mutations that affect electrochemical coupling affect sensing currents as well. During the return of the VSD to the resting state, OFF-sensing currents display a slower kinetic than those observed for ON-sensing currents during activation (Figure 1). To explain this observation, it has been proposed that the VSD controls the binding of the Phospholipid Binding Motif (PBM) to the membrane, which, in turn, controls catalytic activity (Villalba-Galea et al., 2009a; Kohout et al., 2010; Lacroix et al., 2011; Hobiger et al., 2012). Therefore, the return of sensing charges must overcome PBM binding to the membrane while in transit to the resting state. More recently, it has been shown that the PBM is likely to bind PI(4,5)P_2_ (Kohout et al., 2010; Villalba-Galea, 2012). Several mutations in the PBM has been identified to disrupts binding, thus, electrochemical coupling (Villalba-Galea et al., 2009a; Kohout et al., 2010; Lacroix et al., 2011; Hobiger et al., 2012). In the presence of some these mutations or when the catalytic domain is deleted, an increase in the speed of OFF-sensing current is observed (Villalba-Galea et al., 2009a; Hobiger et al., 2012). Conversely, when a mutation causes the “trapping” of the catalytic domain on the membrane, the return of the S4 segment to the resting state is much slower. This has been shown in TE-VCF recordings from Ci-VSP bearing a mutation in catalytic domain where Aspartate 331 is replaced to an alanine (D331A; Kohout et al., 2010). Taken together, these observations clearly suggest that as the VSD controls the catalytic domain, this latter one influences the electrical activity of the sensor. Whether the modulation of electrical activity the consequence of electromechanical coupling or whether there is an explicit feedback mechanism for regulation of the VSD remains to be determined.

## The Voltage Sensing Domain

**Figure 2:**
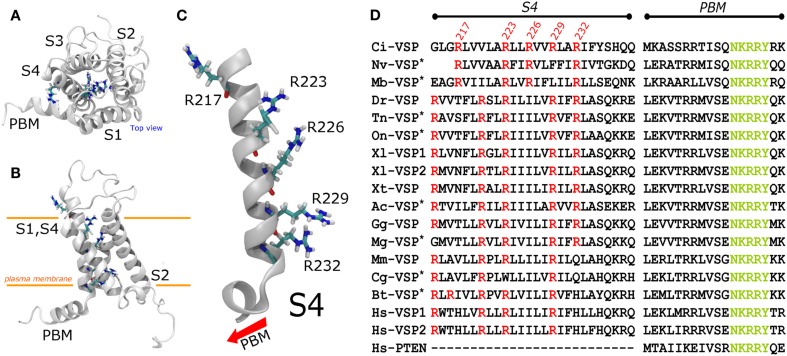
**A structural model for the Ci-VSP VSD was generated using the package MODELER and the structure of the chimeric potassium channels Kv1.2–2.1 (2R9R), subjected to minimization and an all-atom simulation for 50 ns using NAMD**. For molecular dynamics simulations, the structure was embedded in a DPPC lipid bilayer (not shown). **(A)** Top view displaying four transmembrane segments (S1–S4) in counterclockwise order. **(B)** Side view of the Ci-VSP VSD model. The S4 segment displays five Arginines. Arginines 223, 226, 229, and 232 (R1–R4) are located in the center of the crevice formed by the packing of the helices. In contrast, Arginine 217 remains outside the crevice pointing toward the lipids. **(C)** S4 segment shown in details. All charged residues point to the center of the crevice (right side), except for R217 which faces the opposite direction. **(D)** Alignment of the S4 segment of VSP from several species. The PBM, particularly, the sequence NKRRY, was used as a reference point. The arginine corresponding to Ci-VSP’s R223 and R229 are the most conserved arginine among the VSP consulted for this review. Those sequences labeled with an asterisk are predicted proteins. The two letter code before “VSP” represent the species. Mb, *Monosiga* *brevicollis* (marine choanoflagellate); Ci, *Ciona* *intestinalis* (sea squirt); Nv, *Nemastotella* *vectensis* (sea squirt); Dr, *Danio* *rerio* (zebrafish); Tn, *Tetraodon* *nigroviridis* (puffer fish); On, *Oreochromis* *niloticus* (tilapia); Xl, *Xenopus* *laevis* (african clawed frog); Xt, *Xenopus* *tropicalis* (frog); Ac, *Anolis* *carolinensis* (lizard; green anole); Gg, *Gallus* *gallus* (chicken); Mg, *Meleagris* *gallopavo* (turkey); Mm, *Mus* *musculus* (mouse); Cg, *Cricetulus* *griseus* (chinese hamster); Bt, *Bos* *taurus* (cow); Hs, *Homo* *sapiens*. To make the nomenclature uniform, the following changes in notation were made: Hs-VSP1 is TPTE2 or TPIP, Hs-VSP2 is TPTE, and Mm-VSP is mTpte.

The N-terminus of Ci-VSP displays four putative transmembrane spanning segments forming a VSD (Murata et al., 2005). This domain is homologous to those found in voltage gated channels (Noda et al., 1986). The fourth putative segment of the VSD of Ci-VSP bears five basic residues which are thought to constitute the main sensing charges of the domain (Figure 2). In the original description of Ci-VSP, the arginine at position 223 (R223) was alluded as the first sensing charge (Murata et al., 2005). Consistently, it is to be noticed that Arginine 217 (R217) is the only arginine in the S4 segment that is not in the canonical every-third residues array like in many VGC (Figure 2; Horn, 2005). However, neutralization of R217 – the outermost extracellular charge – by mutation to a glutamine (R217Q) shifts the voltage dependence of the VSD about 50 mV toward negative potentials (Dimitrov et al., 2007; Kohout et al., 2008). These observations have prompted the idea that R217 may be the first sensing charged residue of the S4 segment (Kohout et al., 2008). Yet, it can be argued that R217 does not participate in voltage sensing and, instead, its charge causes an electrostatic bias in the effective electric field across the VSD. Thus, whether R217 is the first sensing charge or whether it shapes the electrical field across the VSD remains elusive.

The next charged residues are located in positions 223, 226, 229, and 232. These positive charges are also carried by arginines (Figure 2). Intriguingly, Ci-VSP is the only example –among the sequences consulted for this review – of a VSP with a S4 segment displaying four arginines in a single every-third-residue array (Figure 2). Using the conserved motif NKRRY in the PBM as reference, sequence alignment of Ci-VSP with other VSPs shows that R229 is one the most conserved residues in the S4 segment residues among VSPs (Figure 2). This suggests that R229 may constitute a critical residue for electrochemical coupling and for structural stability of the VSD. For Ci-VSP, substitution of residues 229 and 232 for glutamine abrogated voltage-dependent catalytic activity and seems to suppress sensing currents (Murata et al., 2005). These observations indicate that these residues are likely involved in electrochemical coupling in Ci-VSP.

**Figure 3:**
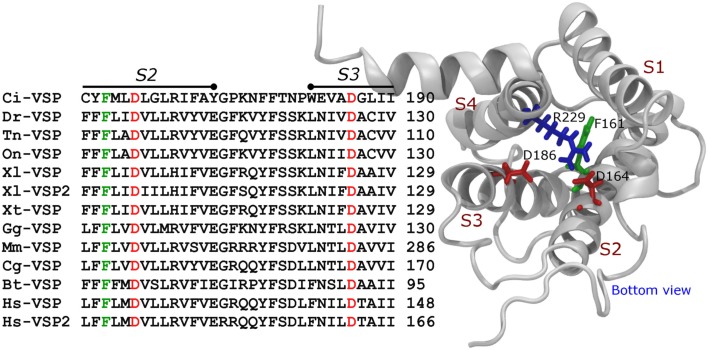
**Aligment of the S2 segment, S2–S3 loop, and S3 segment of VSPs**. The bottom parts of the S2 and S3 segment contain three of the most conserved residues in VSPs. These are the equivalents to F161, D164, and D186 in Ci-VSP. These residues are also found in VGC (see text).

Modeling of the activated VSD of Ci-VSP built based on the crystal structure of the chimeric potassium channel Kv1.2/2.1 (Long et al., 2007) shows that R229 is located in proximity to two negatively charges residues, Aspartate 164 (D164) and Aspartate 186 (D186; Figure 3). In *Shaker*, K374 is critical for structural stability and is likely to interact with Glutamate 293 and Aspartate 316 in the S2 and S3 segments, respectively (Papazian et al., 1995; Tiwari-Woodruff et al., 1997; Khalili-Araghi et al., 2010). Likewise, D164 and D186 are conversed in all known VSPs sequence consulted for this review, suggesting that *Shaker’s* K374 and Ci-VSP’s R229 may play similar roles. Although experimental evidences are to be provided, based on the predicted similarities between these VSD structures, it is likely that R229 is part of a network involving D164 and D186. It is important to emphasize that these interactions might be established at positive potentials, since the model for Ci-VSP shown here was based on the active (maybe relaxed) structure of the Kv1.2–2.1 chimeric channel (Long et al., 2007). Evidently, the accuracy of these predictions is intimately dependent on the initial sequence alignment used for the model construction.

Another residue, Phenylalanines 161 (F161) in Ci-VSP, is also conserved among VSPs (Figure 3). This residue seems to be homologous to F290 in *Shaker*, which is known as the “gating charge transfer center” and constitute the core of the so-called “hydrophobic plug” in the VSD of VGC (Tao et al., 2010; Lacroix and Bezanilla, 2011; Pless et al., 2011). However, it is intriguing that mutations of F161 have little effect on the *Q−V* relationship of Ci-VSP (Lacroix and Bezanilla, 2012) suggesting that the putative “hydrophobic plug” in Ci-VSP may be architecturally different than in VGC.

## Relaxation of Ci-VSP

An fascinating property of Ci-VSP is that the voltage dependence of sensing currents shifts toward negative voltages when the membrane potential is held antipolarized (Villalba-Galea et al., 2008, 2009a). This phenomenon, known as relaxation, has been proposed to occurs following a voltage-independent transition from the Active state (Figure 1C; Villalba-Galea et al., 2008, 2009a). Although the origin of relaxation remains unknown, it has been suggested that one plausible mechanism for it involves local remodeling of the S4 segment. Particularly, a secondary structure transition of the S4 segment from a 3_10_ helix to an α-helix (Villalba-Galea et al., 2008). As known, the carbonyl group of a residue in a 3_10_ helix interacts, via hydrogen bonding, with the amide group of the following third residue. This is different than α-helices in which the equivalent interaction is established with the fourth residue instead. Consequently, as stated by Vieira-Pires and Morais-Cabral (2010), “a 3_10_ helix is more tightly wound, longer, and thinner than an α-helix with the same number of residues.”

A transition in the S4 segment from a 3_10_ to an α-helix can be seen as a “local” mechanism. However, relaxation seems to compromise the entire S4 segment as shown from FRET-based optical recording using the Voltage-Sensitive Fluorescence Protein (VSFP) 2.3 (Villalba-Galea et al., 2009b). To know, VSFPs (Sakai et al., 2001; Baker et al., 2007; Dimitrov et al., 2007; Lundby et al., 2008) and similar construct, such as *Nema*, *Zahra*, and *Zahra* 2 (Baker et al., 2012), are artificial proteins built by fusing fluorescence proteins to the C-terminus of a VSD. Detailed analysis by Akemann et al. (2009) has confirmed that optical signals from VSFP 2.3 report conformational changes related to VSD relaxation. Thus, it can be argued that relaxation may arise from rearrangements of the entire VSD to energetically satisfy the new position of the S4 segment after activation. If this is the case, relaxation can be seen as a “global” mechanism.

The structures of several six-transmembrane domain channels display 3_10_ helices in their S4 segments (Long et al., 2007; Clayton et al., 2008; Payandeh et al., 2011). In the structure of the chimeric potassium channel Kv1.2–2.1, a 3_10_ helix is found in the bottom the S4 segment extending from the fourth (R4) to the sixth (R6) arginines (Long et al., 2007). In the case of the NavAb, a member of the NaChBac family isolated from the bacterium *Arcobacter* *butzleri*, a 3_10_ helix extends along the S4 including the four arginines of this segment (Payandeh et al., 2011). Similarly, the MlotiK1 structure shows its charge-less S4 segment displaying a five-turn 3_10_ helix (Clayton et al., 2008). Because the existence and stability of 3_10_ helices depend on the packing and the interaction with other regions (Vieira-Pires and Morais-Cabral, 2010), these observations grant the possibility that S4 segment could be packed as a 3_10_ helix in the resting state. In support of this idea, several molecular dynamics studies of isolated VSDs suggest that the S4 segment rests as a 3_10_ helix (Bjelkmar et al., 2009; Khalili-Araghi et al., 2010; Schwaiger et al., 2011). In fact, it has been proposed that the S4 segment moves more readily when packed in a 3_10_ helix when compared to an α-helix (Bjelkmar et al., 2009; Schwaiger et al., 2011). In summary, these studies seem to conclude that, indeed, the property of being “tightly wound, longer, and thinner” is likely to be more energetically favorable for voltage sensing. Thus a combination of “local” and “global” events can account for relaxation.

## Voltage Clamp Fluorometry and Relaxation

Using the TE-VCF technique, it has been shown that the quenching of tetramethylrhodamine-maleimide (TMRM) attached to a Cysteine replacing *Glycine* 214 (G214) on the top of the S4 segment is sensitive to conformational changes in the VSD (Kohout et al., 2008). Detailed analysis of fluorescence recording using Cut-Open Voltage Clamp Fluorometry (CO-VCF) revealed that TMRM quenching reports two distinct conformational changes during VSD activation (Villalba-Galea et al., 2008). The first (fast) component is correlated with the movement of the sensing charges, constituting about 40% of the change in fluorescence observed (pulsing to +80 mV for 2 s). The second component (slow) is correlated with the settling of relaxation of the VSD as estimated by electrophysiology. This second component develops after sensing currents have faded, suggesting that the conformational changes responsible for this quenching component are not caused by voltage-dependent transitions (Villalba-Galea et al., 2008). Combining both observations, it was concluded that this significant fraction of the fluorescence quenching signal emerge from conformational changes involved in relaxation (Villalba-Galea et al., 2008).

The structure of the chimera Kv1.2–2.1 is regarded as being in the active state. However, since there is no electrical field imposed across the VSD during crystallization, this leads to the possibility that the VSD in structure is in a relaxed-like state and that the top half of the S4 segment displays an α-helix that forms after relaxation (Villalba-Galea et al., 2008). Furthermore, it has been proposed from molecular dynamics studies that the movement of the S4 segment produces little changes in the shape and intensity of the electrical fields across the VSD during deactivation (Delemotte et al., 2011). Extrapolating from this observation, it can be argued that the movement of the sensor during activation occurs before any secondary structure change takes place.

Based on VCF studies in *Shaker*, it has been proposed that the depolarization-induced movement of the S4 segment involves a rotation of the helix along its axis (Tombola et al., 2006; Pathak et al., 2007). The possibility of a transition from a 3_10_ to an α-helix occurring after sensing (gating) currents inexorably leads us to a simple minded question: Is it possible that the apparent rotation of the S4 is the consequence of the unwinding of the 3_10_ helix? In the case of Ci-VSP, this question has not been answered yet. However, it has been shown that TMRM-labeling at position 208 (a glutamine to cysteine substitution in the S3–S4 loop) yields a fluorescence signals displaying a biphasic behavior. In this case, antipolarization causes an initial dequenching of the fluorophore followed by a slow quenching beyond the resting value (Kohout et al., 2010). This observation suggests that the TMRM attached to position 208 “visits” two different environments causing this differential quenching and – based on what is know for *Shaker* (Pathak et al., 2007) – are consistent with the idea that the top of the S4 segment is rotating. As before however, it can be argued that the unwinding of S4 segment after activation could produce a similar fluorescence signature. These possibilities are not exclusive and can not be ruled out with the reported evidences. So, further confirmation is needed.

It is noteworthy that alternative models for the movement of the S4 segment, such as the “paddle” model (Jiang et al., 2003; Ruta et al., 2003; Long et al., 2007) and the “helical-screw” model (Guy and Seetharamulu, 1986) have not been ruled out. Yet, the model depicted here is more in tune with a third of “the three Major Schools” – as referred to by Borjesson and Elinder (2008) – in which the core of the VSD forms water-filled crevices.

Noteworthy, recent work from the Elinder Lab (Henrion et al., 2012) shows that, in *Shaker*, the S4 segment moves respect to the S3b segment and not with it, suggesting that the paddle model is inadequate. Also, a number of recent theoretical studies support this finding (Pathak et al., 2007; Khalili-Araghi et al., 2010; Delemotte et al., 2011; Schwaiger et al., 2011; Jensen et al., 2012; Yarov-Yarovoy et al., 2012).

Thus far, as reported by fluorescence measurements, the electrically driven movement of the S4 segment seemingly leads to the unwinding (or rotation) of the S4 segment outermost section. This notion might suggest that conformational changes of the VSD during relaxation are confined to the top part of the S4 segment. This implies that relaxation consists of a “local” rearrangement of this segment. However, fluorescence recording from VSFP2.3 expressed in *Xenopus* oocytes argues otherwise (Villalba-Galea et al., 2009b). Reiterating, VSFP2.3 is a member of a genetically encoded optical probes for membrane potential built by attaching fluorescent proteins – or tandem of them – to the C-terminus of a VSD (Sakai et al., 2001; Baker et al., 2007; Dimitrov et al., 2007; Lundby et al., 2008). In the case of VSFP2.3, a tandem of Cyan- and Yellow-Fluorescent Proteins (CFP and YFP, respectively) replaces the catalytic domain of Ci-VSP. Using CO-VCF, fluorescence recordings from this probe have shown that the optical signals are correlated with sensing charge movement and relaxation in a similar fashion than fluorescence signals from TMRM when covalently attached to the other end of the S4 segment (Villalba-Galea et al., 2009b). These observations clearly indicate that relaxation is transmitted along the entire S4 segment, rather to be a “local” event. Thus, it is possible that relaxation involves conformational changes in other regions of the VSD in addition to the S4 segment. If proven, this will make relaxation a “global” phenomenon. Models for the resting state of Kv channels show a rearrangement of the VSD involving all transmembrane segments respect the active state (Pathak et al., 2007; Khalili-Araghi et al., 2010; Yarov-Yarovoy et al., 2012). Therefore, it is very likely that relaxation encompasses conformational changes in the entire VSD. Further investigations of this matter will provided a better understanding of the dynamic of voltage sensor. This facet in the activity of Ci-VSP has constituted – and remains to be – a great tool in doing so.

## Final Remarks

Why VSPs operate at positive potentials remains unclear – such is their physiological role. However, membrane potential is not the only parameter determining the activity of VSPs – at least for Ci-VSP. Under physiological conditions, operation of VSPs could be tightly regulated by phosphatidylinositol 4,5-bisphosphate [PI(4,5)P_2_] beyond activation by PBM binding. Electrochemical coupling is regulated by this lipid (Kohout et al., 2010) and, in turn, Ci-VSP and other VSPs, use this signaling molecule as one of their main catalytic substrates (Murata et al., 2005; Murata and Okamura, 2007; Halaszovich et al., 2009; Kohout et al., 2010; Ratzan et al., 2011). Combining these observations, it can be speculated that VSPs could function as homeostatic regulators for the concentration of PI(4,5)P_2_, where the combination of electrical activity and PI(4,5)P_2_, are dynamically tuning phosphatase activity.

## Conflict of Interest Statement

The author declares that the research was conducted in the absence of any commercial or financial relationships that could be construed as a potential conflict of interest.
