# Corrigendum: Voltage-controlled enzymes: the new Janus Bifrons

**DOI:** 10.3389/fphar.2015.00109

**Published:** 2015-05-29

**Authors:** Carlos A. Villalba-Galea

**Affiliations:** Department of Physiology and Biophysics, Virginia Commonwealth University School of MedicineRichmond, VA, USA

**Keywords:** VSD relaxation, Ci-VSP, voltage-sensitive phosphatases, sensing currents, hysteresis

Due to an error, there is an mistake in panel C of the original version of Figure 1. The correct version of Figure 1 is presented below. The figure legend has been updated to improve readability, but the message remains unaltered.

**Figure 1:**
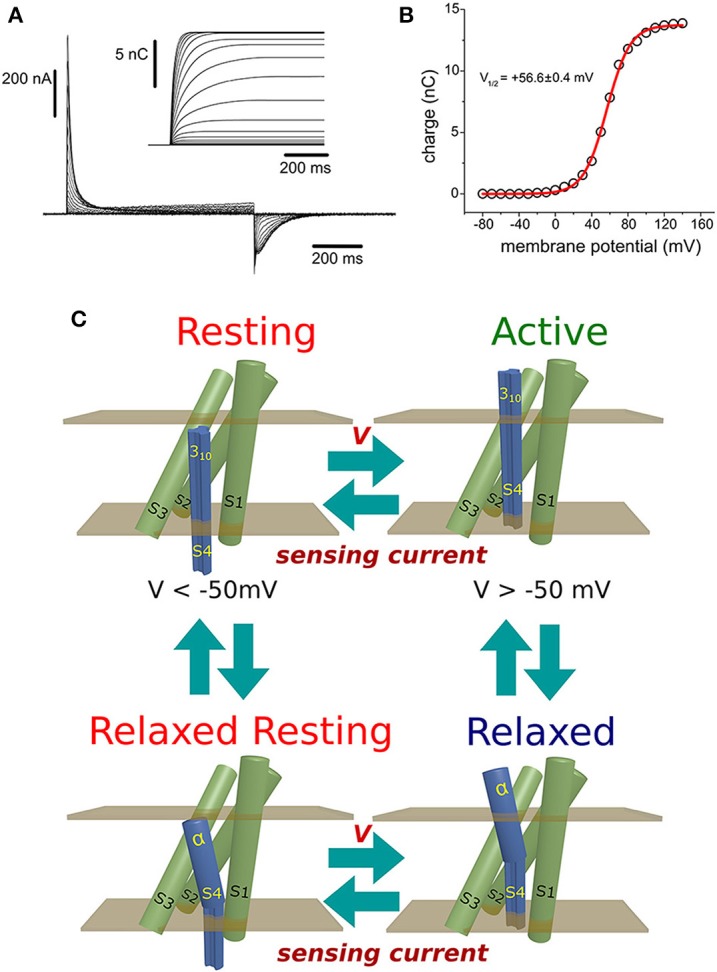
**(A)** Ci-VSP-C363S sensing currents recorded from *Xenopus* oocytes using the Cut-Open Voltage-Clamp technique (Taglialatela et al., 1992; Stefani and Bezanilla, 1998). The holding potential (HP) was set to −60 mV, and ON-sensing currents were evoked by 800 ms-test pulses ranged from −80 mV to +140 mV. OFF-sensing currents were recorded at −60 mV. Numerical integration of the ON-sensing currents (inset) was performed with a package developed by the author using the programming language Java. **(B)** The calculated net charges were plotted against the voltage applied during the corresponding test pulse. The charge (Q) vs. potential (V) relationship was fitted to a Boltzmann distribution (see text). For this particular example, the fitted half-maximum potential was +56.6 ± 0.4 mV. **(C)** Minimum scheme describing the electrical behavior of the voltage sensing domain of Ci-VSP: At potentials below −50 mV, the VSD resides with high probability in the resting state. Upon changing the membrane potential to more positive voltages, sensing currents were produced as sensing charges moves down the electrical gradient, leading the VSD into the active state. If the membrane potential is above +50 mV, a secondary, voltage-independent transition is observed. During this process, called relaxation (see text), the relaxed state is populated. Transitions between the resting and active state may occur while the S4 segment is in a 3_10_ helix conformation. However, transit into the relaxed states may be accompanied by a transformation of the upper part of the S4 segment into an α-helix. Repolarization of the plasma membrane drives the return of the VSD to the resting state. This transition is achieved through a hypothetical relaxed-resting state.

## Conflict of interest statement

The author declares that the research was conducted in the absence of any commercial or financial relationships that could be construed as a potential conflict of interest.

## References

[B1] Stefani E. Bezanilla F. (1998). Cut-open oocyte voltage-clamp technique. Methods Enzymol. 293, 300–318. 9711615 10.1016/s0076-6879(98)93020-8

[B2] Taglialatela M. Toro L. Stefani E. (1992). Novel voltage clamp to record small, fast currents from ion channels expressed in Xenopus oocytes. Biophys. J. 61, 78–82. 1311612 10.1016/S0006-3495(92)81817-9 PMC1260224

